# O022: Development of an electronic dashboard to assist surveillance

**DOI:** 10.1186/2047-2994-2-S1-O22

**Published:** 2013-06-20

**Authors:** S Wallace, N Damani

**Affiliations:** 1 Infection Prevention and Control, Southern Trust, Craigavon, UK

## Introduction

Collection and timely feedback of process and outcome surveillance is one of the most challenging tasks faced by the Infection Prevention and Control (IPC) team. The aim of our project was to develop a system to ensure timely communication of surveillance information to both clinical and non-clinical teams using an electronic dashboard.

## Methods

Our hospital developed in house e-reporting tools for use on our IT network. The system was created using Visual Basic programming and utilised existing word processing and database software. Each PC terminal in clinical areas was granted access to the e-reporting forms. Staff were provided with brief 15 minute training sessions detailing how to use the system.

## Results

Since the introduction of the e-dashboard system we have been able to host all our information in one location, with regular updates, allowing ease of access for all staff. Staff can now compare their performance against other departments and by staff grouping. This has provided a ‘nudge’ effect and their compliance with IPC practices has gradually improved as they do not want to feature as an outlier. In addition to this, the benefit of the online availability of the information dramatically reduced the need for sending e-mails, thus saving time and reducing pressure on the Trust’s IT network infrastructure.

## Conclusion

Since the introduction of the electronic dashboard, the hospital within our Trust has seen a substantial improvement in communicating of both process and outcome surveillance information to both clinical and non-clinical teams. As a result of this feedback we have seen a substantial improvement in compliance with process surveillance for example, the hand hygiene return rates have risen from pre-intervention 62% to 82% post intervention. As a result of improved compliance in other areas (environmental cleanliness, antibiotic prescribing, commode cleaning etc.) we have seen over 70 % reductions in *C.difficile* and MRSA bacteraemia infections. The system used can be replicated by any hospital with minimal resources when compared to commercial systems that require costly support and contracts to meet local needs. The system also allows the release of IPC time crucial in low resource settings where resources are constrained.

## Disclosure of interest

None declared.

